# Supplementary material for: CRISPR-dCas13d-based deep screening of proximal and distal splicing-regulatory elements
Source: Nat Commun. 2024 May 7;15:3839. doi: 10.1038/s41467-024-47140-8 (PMC11076525; doi:10.1038/s41467-024-47140-8)
Supplement: Supplementary file 1 — Supplementary Information [file 41467_2024_47140_MOESM1_ESM.pdf]

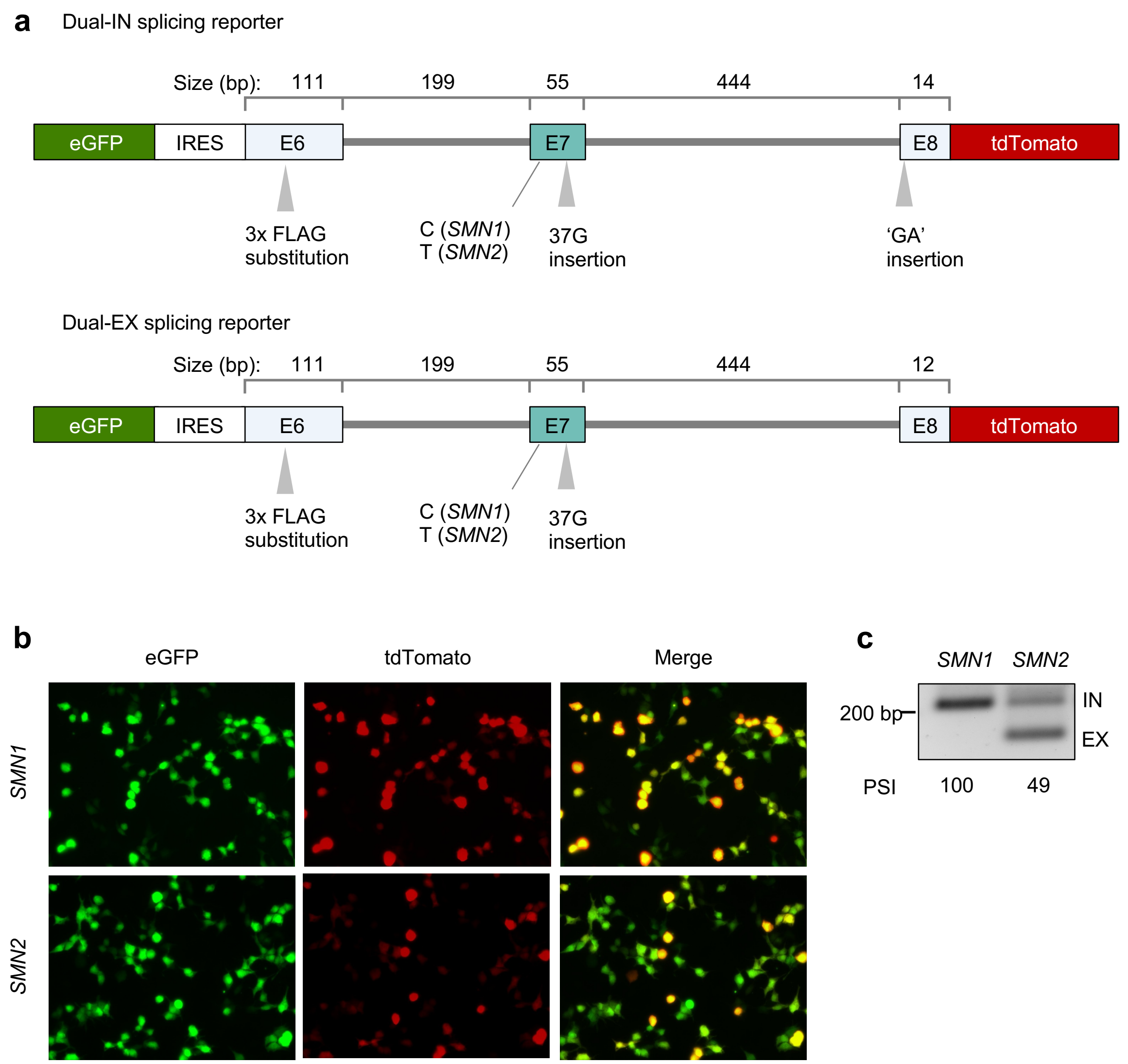

Supplementary Fig. 1: Details of the dual color *SMN1/2* splicing reporter constructs. a, Schematic illustrating the design of the Dual-IN (top) and Dual-EX (bottom) *SMN2* splicing reporter constructs. Several modifications were incorporated, including the addition of a 3x FLAG to exon 6, a 'G' nucleotide insertion at position 37 of exon 7 (originally 54 nt) to remove the natural in-frame stop codon and make it a frame-shifting exon, and an insertion of 'GA' at the beginning of exon 8 to shift tdTomato in-frame when exon 7 is included. The *SMN1* splicing reporter was similarly constructed for comparison and included the T-to-C transition at position 6 of exon 7. **b,** Expression of the Dual-IN *SMN1* and *SMN2* splicing reporters in HEK293T cells after transfection, as measured by eGFP and tdTomato fluorescence. **c,** RT-PCR validation of exon 7 splicing in *SMN1* and *SMN2* splicing reporters. A representative gel image of splicing products is shown at the bottom with the quantification of exon inclusion level (percent spliced in, or PSI Ψ).

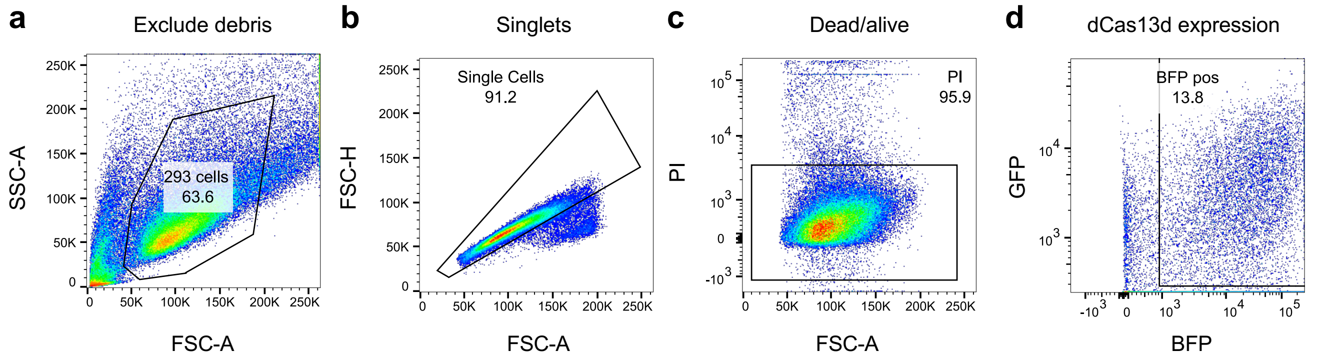

Supplementary Fig. 2: Gating scheme for cells transfected with individual gRNAs. Related to Fig. 2d in the main text. Cells were co-transfected with plasmids expressing the Dual-IN *SMN2* splicing reporter, dCas13d-BFP, and individual gRNAs. **a,b**, Initial forward scatter area and side scatter area (FSC-A vs. SSC-A, a), and FSC-A vs. FSC-height (FSC-H, b) to gate for cells and singlets. **c**, Propidium iodide stain for dead/alive cells. **d**, BFP and eGFP-positive gates for cells expressing dCas13d-BFP and the splicing reporter.

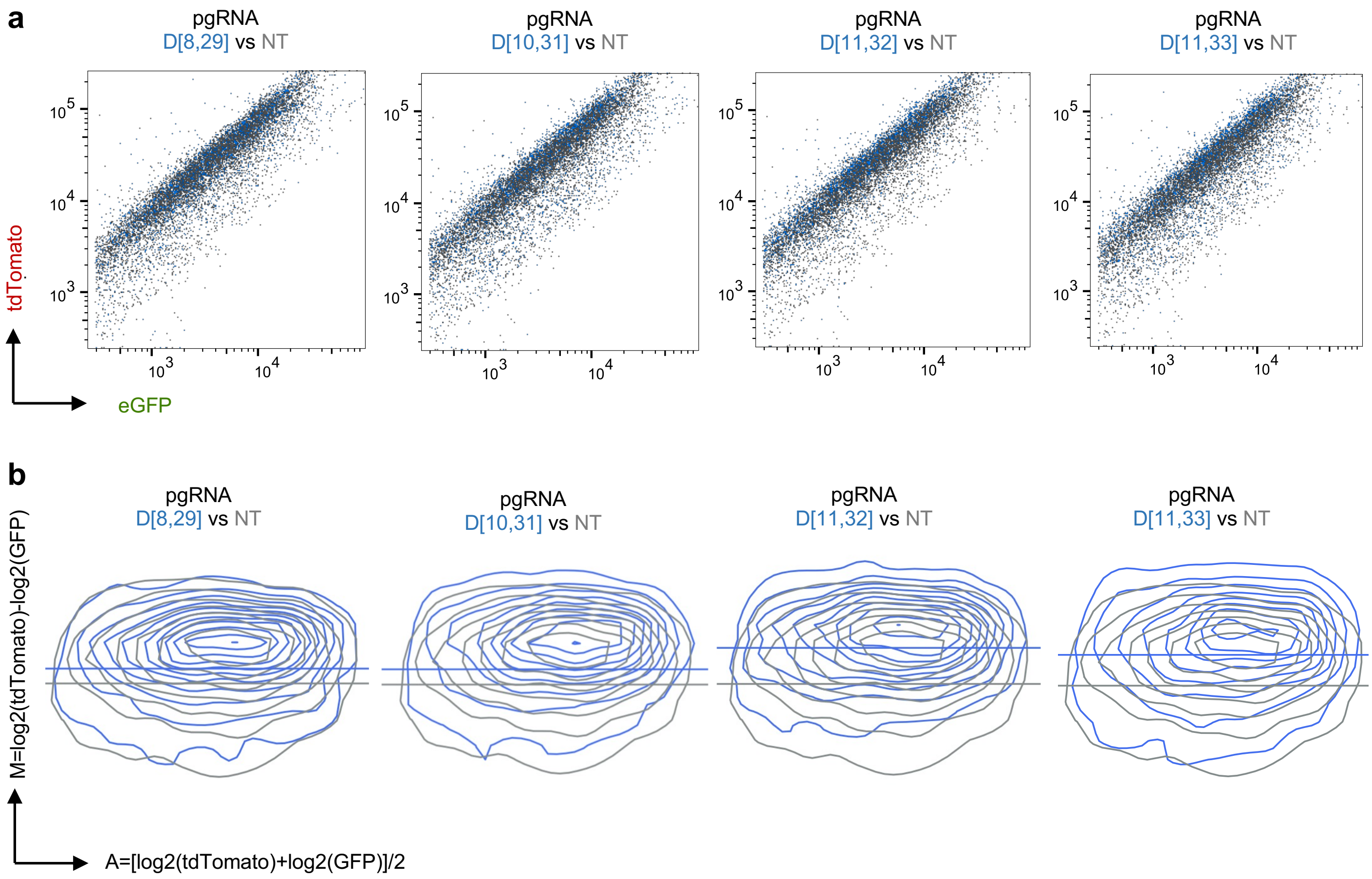

Supplementary Fig. 3: FACS and MA contour plots for additional individually transfected gRNAs. Related to Fig. 2d,e in the main text. **a,** FACS plots. **b,** MA-contour plots. See legends of Fig. 2d for more details.

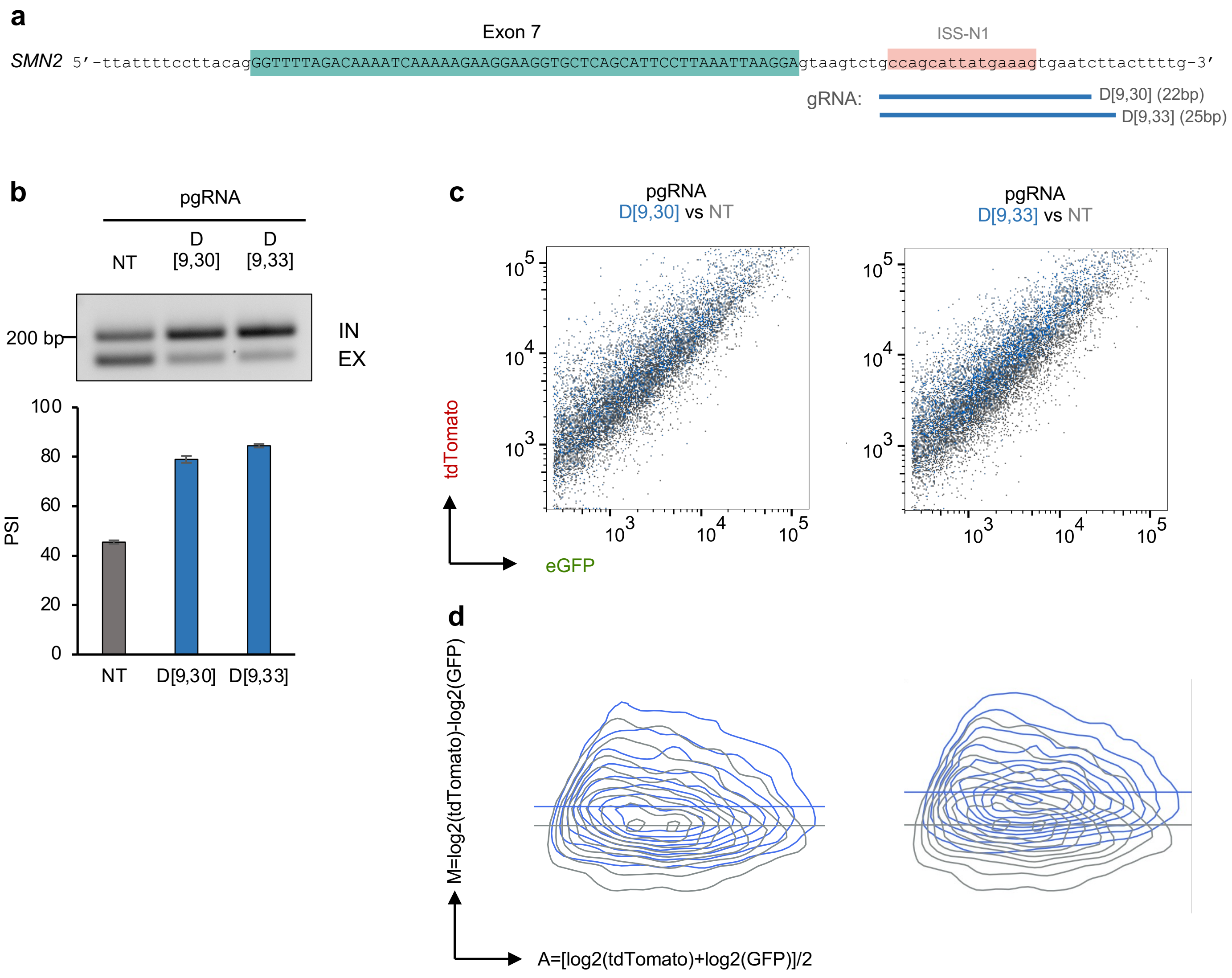

Supplementary Fig. 4: Splicing-modulatory effects of gRNAs of different spacer lengths. a, Schematic illustrating splicing-enhancing gRNAs (blue) of sizes 22-nt and 25-nt targeting the intronic splicing silencer element ISS-N1 (region shaded in red). Each gRNA was transfected along with the Dual-IN *SMN2* splicing reporter and dCas13d-BFP. NT=non-targeting control gRNA. **b,** Representative gel image of RT-PCR products (top) with the quantification of exon 7 inclusion shown at the bottom (N=2). Error bars represent SEM. **c,d,** Quantification of exon 7 inclusion using tdTomato/eGFP fluorescence as shown in FACS plots (c) and MA contour plots (d). FACS/MA plot datasets contain ≥5,000 BFP-positive events per sample.

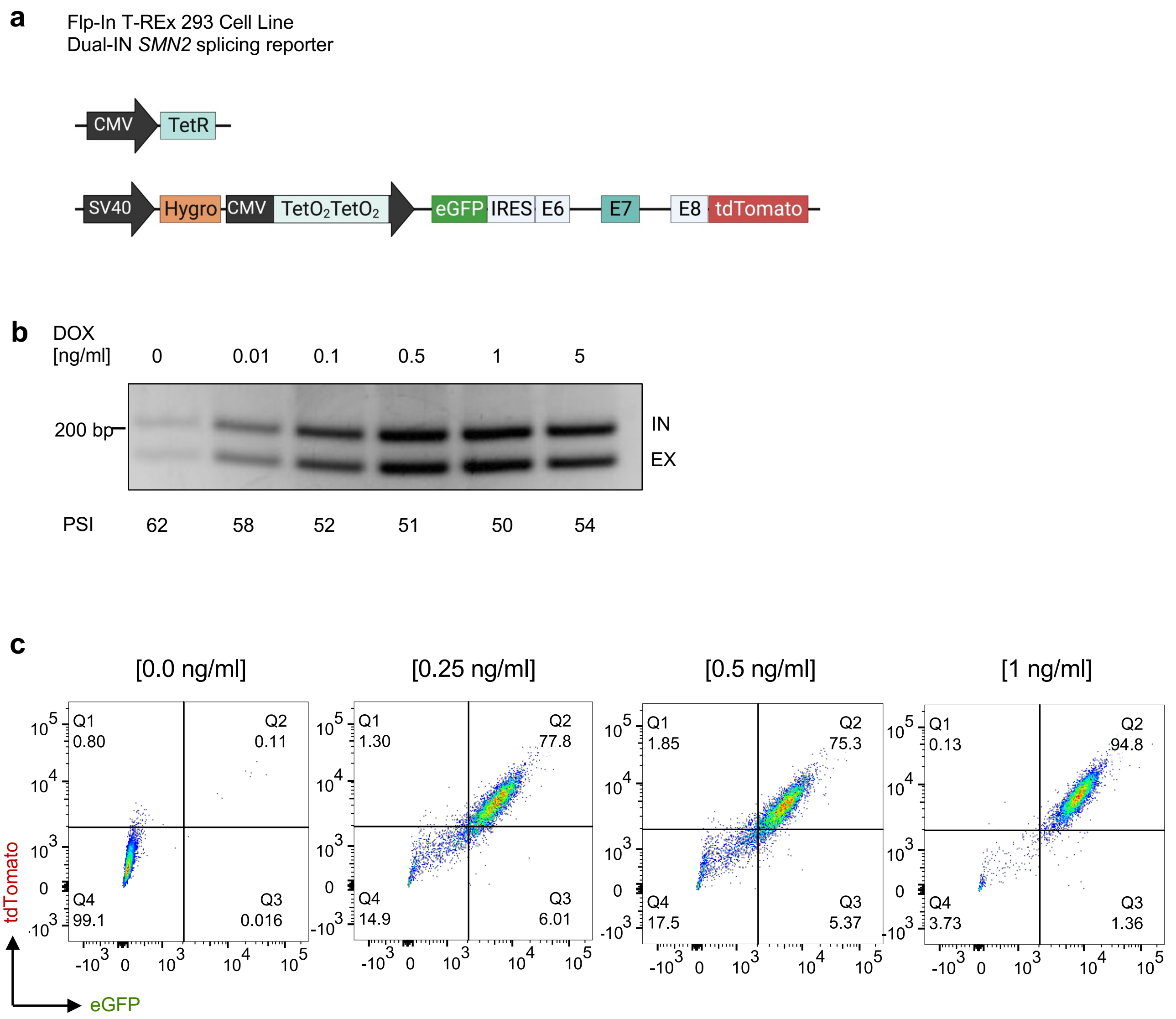

Supplementary Fig. 5: Validation of the DOX-inducible Dual-IN *SMN2* cell line. a, Schematic representation of cell line containing DOX-inducible Dual-IN *SMN2* splicing reporter. **b,** RT-PCR analysis of exon 7 inclusion of the monoclonal DOX-inducible Dual-IN *SMN2* cell line used for screening. Varying concentrations of DOX [0, 0.01, 0.1, 0.5, 1, 5 ng/mL] were tested. A representative gel image of the RT-PCR product is shown with quantification of exon 7 inclusion at the bottom. **c,** The monoclonal cell line was sorted 48 hours after the addition of DOX at varying concentrations. FACS data contains ~6,000 events per plot.

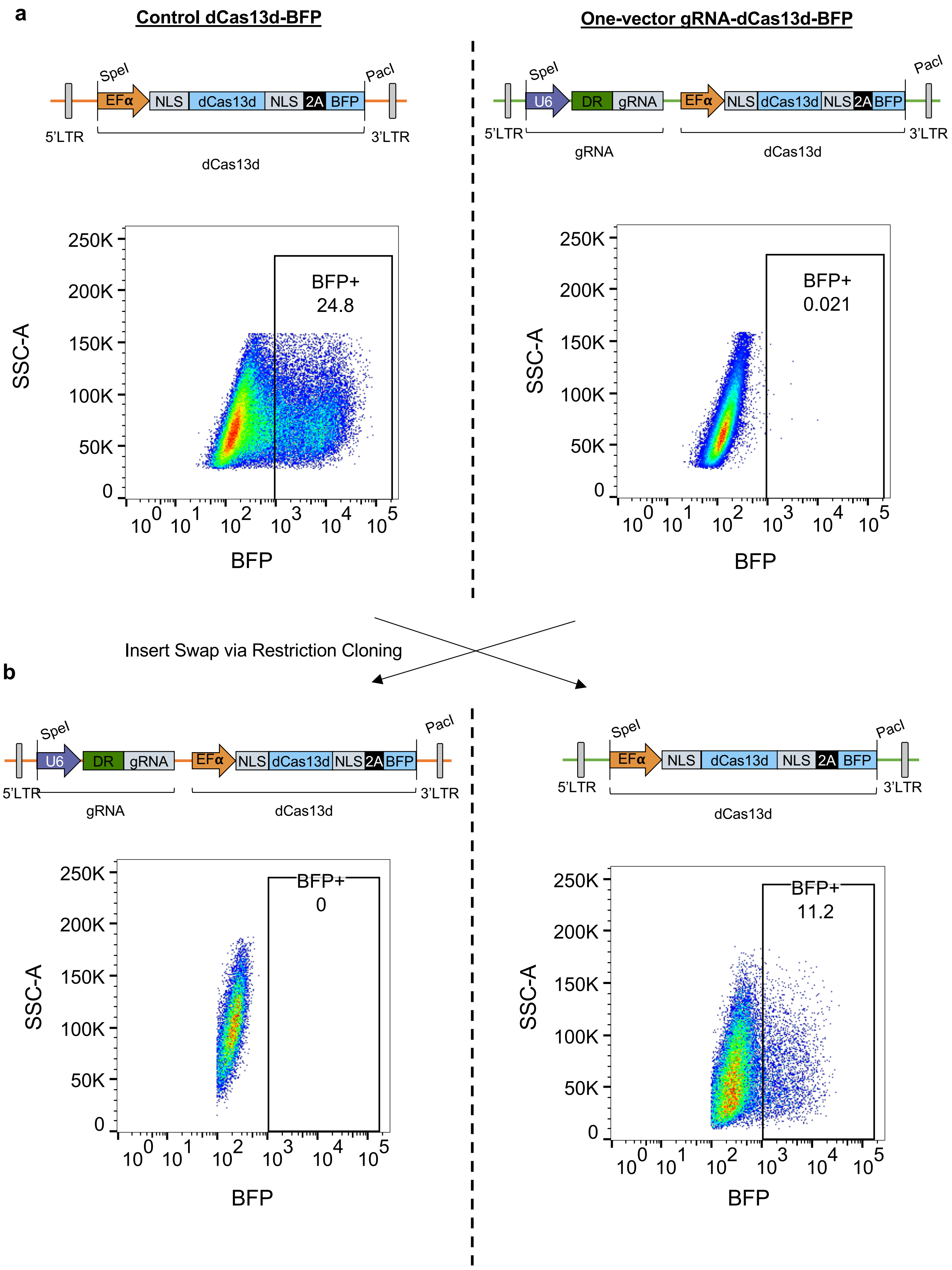
a
Control dCas13d-BFP
One-vector gRNA-dCas13d-BFP
SpeI
PacI
EFα
NLS
dCas13d
NLS
2A
BFP
5'LTR
3'LTR
U6
DR
gRNA
BFP+
24.8
BFP+
0.021
SSC-A
BFP
250K
200K
150K
100K
50K
0
Insert Swap via Restriction Cloning
b
BFP+
0
BFP+
11.2

Supplementary Fig. 6: One-vector gRNA-dCas13d-BFP lentiviral construct produces inefficient lentivirus packaging. a, A one-vector gRNA-dCas13d-BFP construct was cloned using the lentiCRISPR v2 backbone (Addgene: 49535) and used for lentivirus packaging. Few cells, if any, express BFP, as evaluated by FACS sorting. The dCas13d-BFP construct was used as a positive control, which did produce a subset of BFP+ cells. **b**, To exclude the possibility that the different lentivirus backbones used in (a) might play a role, we swapped the inserts by restriction cloning. The gRNA-dCas13d-BFP construct cloned into the previously efficient backbone resulted in a lack of BFP+ cells, while the dCas13d-BFP construct cloned into the new backbone resulted in a subset of BFP+ cells.

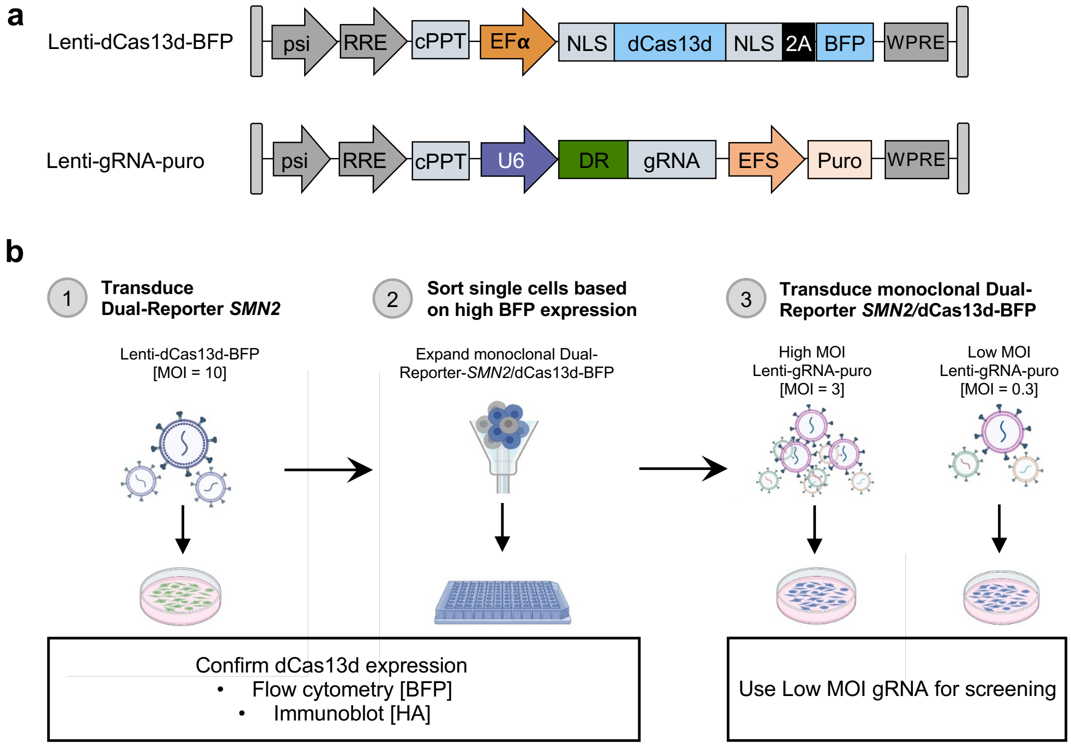

Supplementary Fig. 7: Construction of Dual-IN *SMN2*/dCas13d-BFP stable cell line for screening using lentivirus gRNA libraries. a, Schematics of lentiviral constructs used to deliver dCas13d-BFP and gRNA-puro to the cell line expressing DOX-inducible Dual-IN *SMN2* splicing reporter. **b,** Cells expressing DOX-inducible Dual-IN *SMN2* splicing reporter were transduced with concentrated dCas13d-BFP lentivirus and sorted 48 hours later to quantify BFP-expression level and to isolate single cells with high BFP expression. The single cells were expanded to obtain a monoclonal Dual-IN *SMN2*/dCas13d-BFP cell line. This cell line was then transduced with the lentiviral construct containing an individual gRNA (or a gRNA library) and the selection marker puromycin. Screening data were generated using a low gRNA MOI (0.3), while a comparison of individually transduced gRNAs used both a high MOI (3) and a low MOI (0.1). Schematic was generated using BioRender.

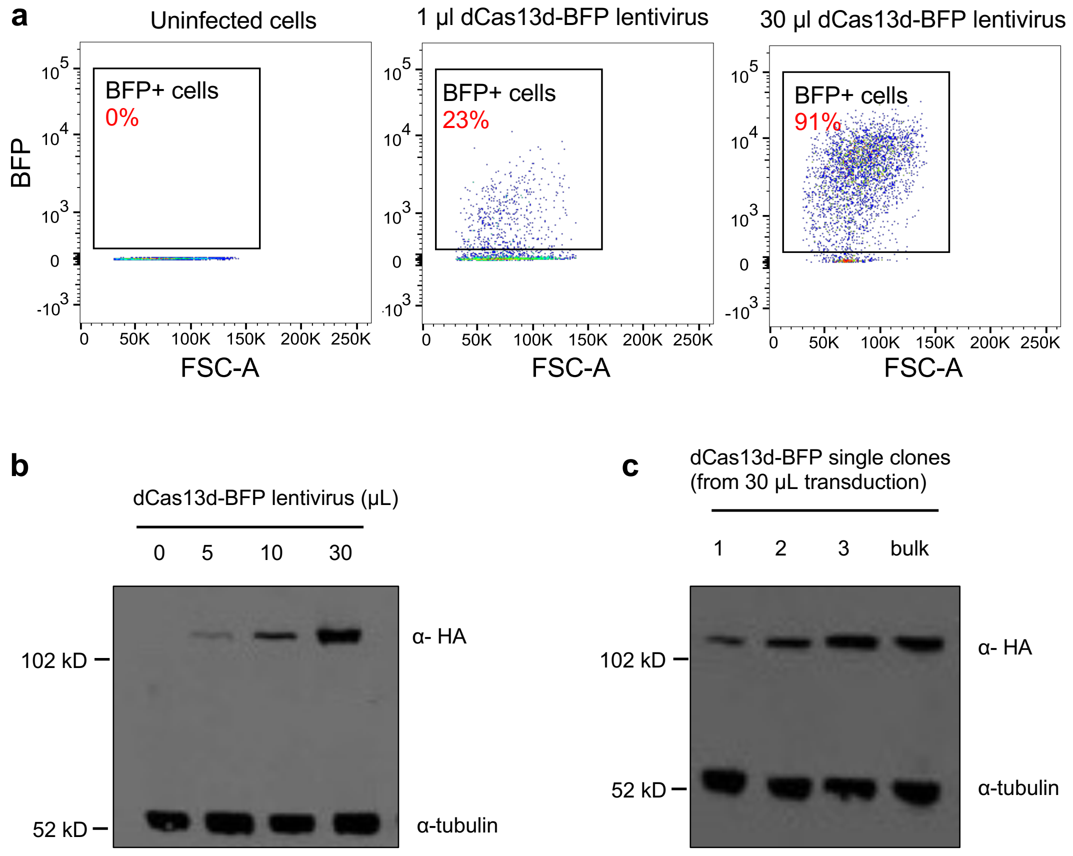

Supplementary Fig. 8: Validation of dCas13d-BFP lentiviral transduction used to obtain monoclonal Dual-IN *SMN2*/dCas13d-BFP stable cell line. a, Dual-IN *SMN2* reporter cells were transduced with varying amounts of concentrated dCas13d-BFP lentivirus (indicated above each FACS plot) and sorted to quantify BFP expression. FACS plot contains ≥2,000 events per sample. **b,** Immunoblot of bulk Dual-IN *SMN2* cell lines after transducing varying amounts of dCas13d-BFP lentivirus. α-HA detects HA-tagged dCas13d. **c,** High BFP-expressing single cells isolated from Dual-IN *SMN2* reporter cell lines transduced with 30uL of dCas13d-BFP lentivirus were expanded into monoclonal cell lines and immunoblots against HA-tagged dCas13d were performed on single clone samples. The parental bulk sample (last lane) was included for comparison.

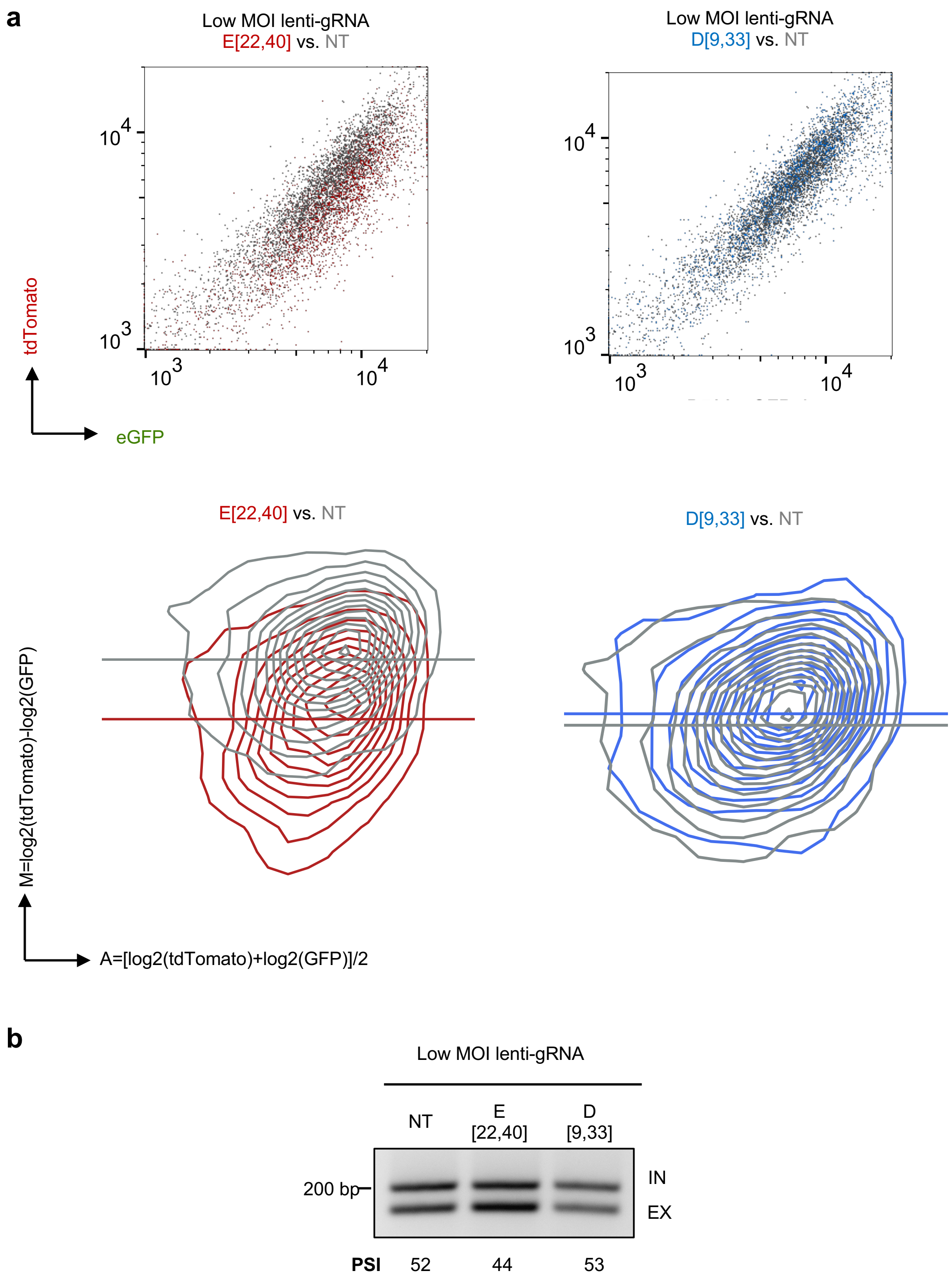

Supplementary Fig. 9: Splicing modulation of individually transduced gRNAs at a low MOI. a, FACS (top) and MA contour plots (bottom) showing Dual-IN *SMN2*/dCas13d-BFP stable cell line transduced with splicing-modulating gRNAs E[22,40] (red) and D[9,33] (blue) with MOI=0.1 to ensure one gRNA per cell. Cells transduced with NT control were overlaid. Datasets were downsampled to 3,200 BFP-positive events per sample. **b,** RT-PCR analysis of exon 7 from BFP-positive cells transduced with gRNAs, with quantification shown below the representative gel image.

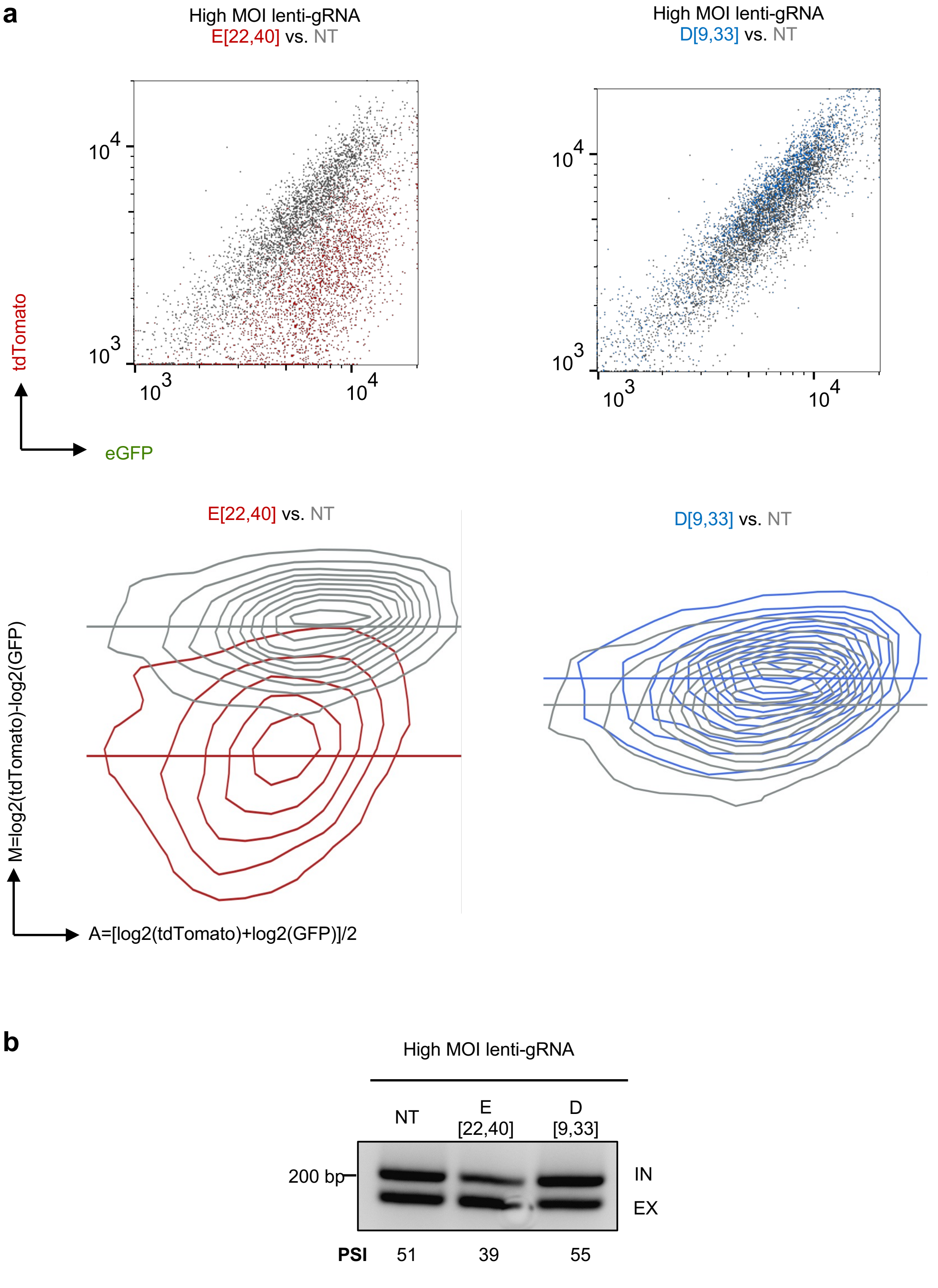

Supplementary Fig. 10: Splicing modulation of individually transduced gRNAs at a high MOI. Similar to Supplementary Fig. 9, except that cells were transduced at a MOI=3.

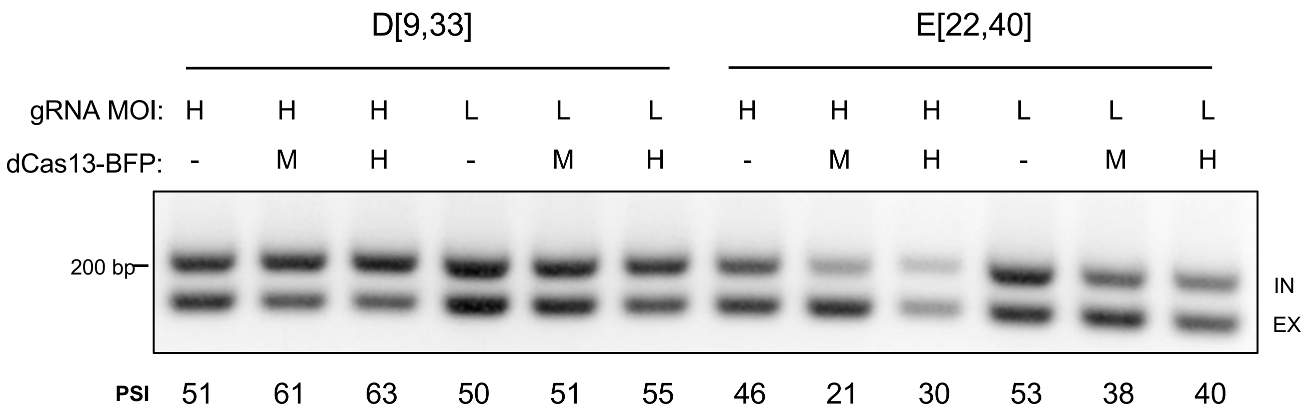

Supplementary Fig. 11: Splicing modulation of individually transduced gRNAs with varying levels of dCas13d-BFP. Dual-IN *SMN2*/dCas13d-BFP cells were transduced with lentivirus expressing individual gRNAs D[9, 33] or E[22, 40] at low (L) or high (H) MOI. For each condition, transduced cells were sorted by FACS based on high (H), medium (M), or no (-) BFP expression. For cells collected in each fraction, *SMN2* exon 7 splicing was analyzed by RT-PCR. A gel image of the splicing products and the quantification of exon 7 inclusion is shown.

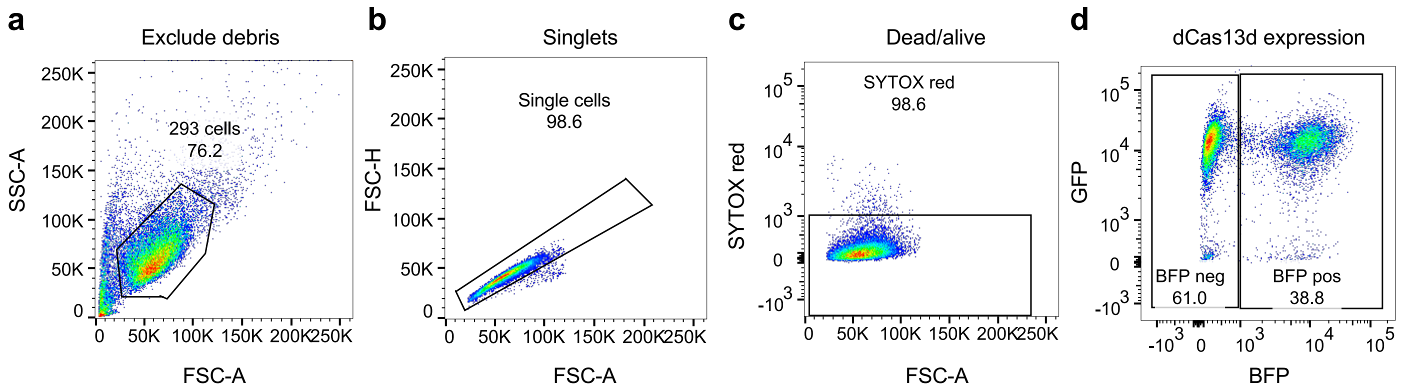

Supplementary Fig. 12: Gating scheme for Dual-IN *SMN2*/dCas13d-BFP cells transduced with lentivirus expressing an individual gRNA or a gRNA library. Cells with stable expression of DOX-inducible Dual-IN *SMN2* splicing reporter and dCas13d-BFP were transduced with lentivirus expressing an individual gRNA or a pooled gRNA library. See Supplementary Figure 2 legends for more details.

a

Dual-IN *SMN2* Samples	Total reads Replicate 1	Perfect gRNA Reads Replicate 1 (%)	Total reads Replicate 2	Perfect gRNA Reads Replicate 2 (%)
Unsorted	9,832,415	7,726,408 (78.6%)	13,085,863	10,729,246 (82.0%)
Top Bin BFP positive	18,223,619	13,704,672 (75.2%)	13,976,985	11,228,267 (80.3%)
Bottom Bin BFP positive	14,074,978	11,298,635 (80.3%)	10,382,100	8,768,692 (84.5%)
gRNAs Not Detected in Unsorted	-	48 (2.5%)	-	59 (3%)

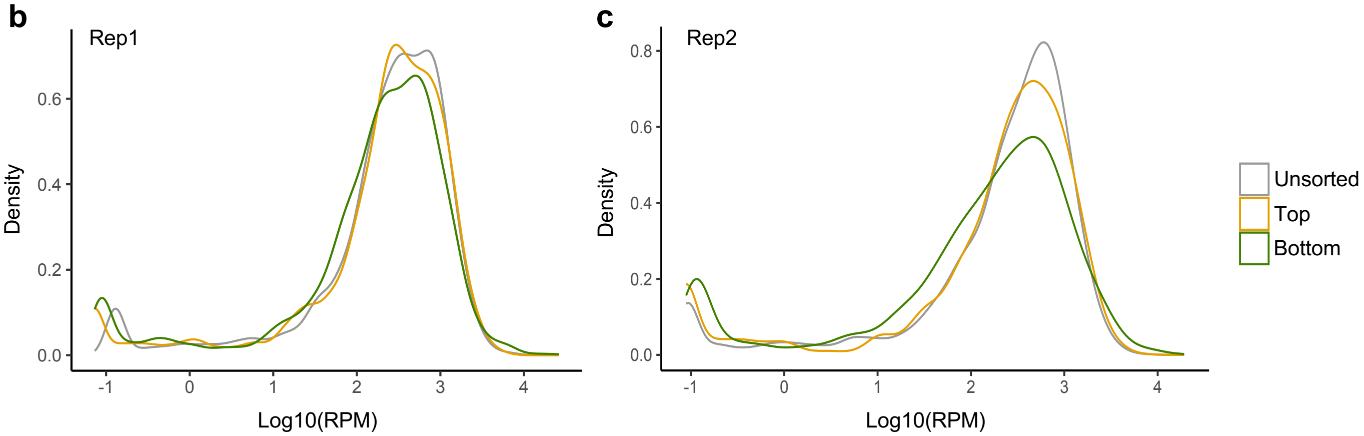

Supplementary Fig. 13: gRNA sequencing statistics for screening of Dual-IN *SMN2* splicing reporter. a, Number of total reads and perfectly matched gRNA reads in each sample from replicate 1 and replicate 2. **b,c,** Distribution of normalized read count, reads per million (RPM) of the gRNA library for replicate 1 (b) and 2 (c).

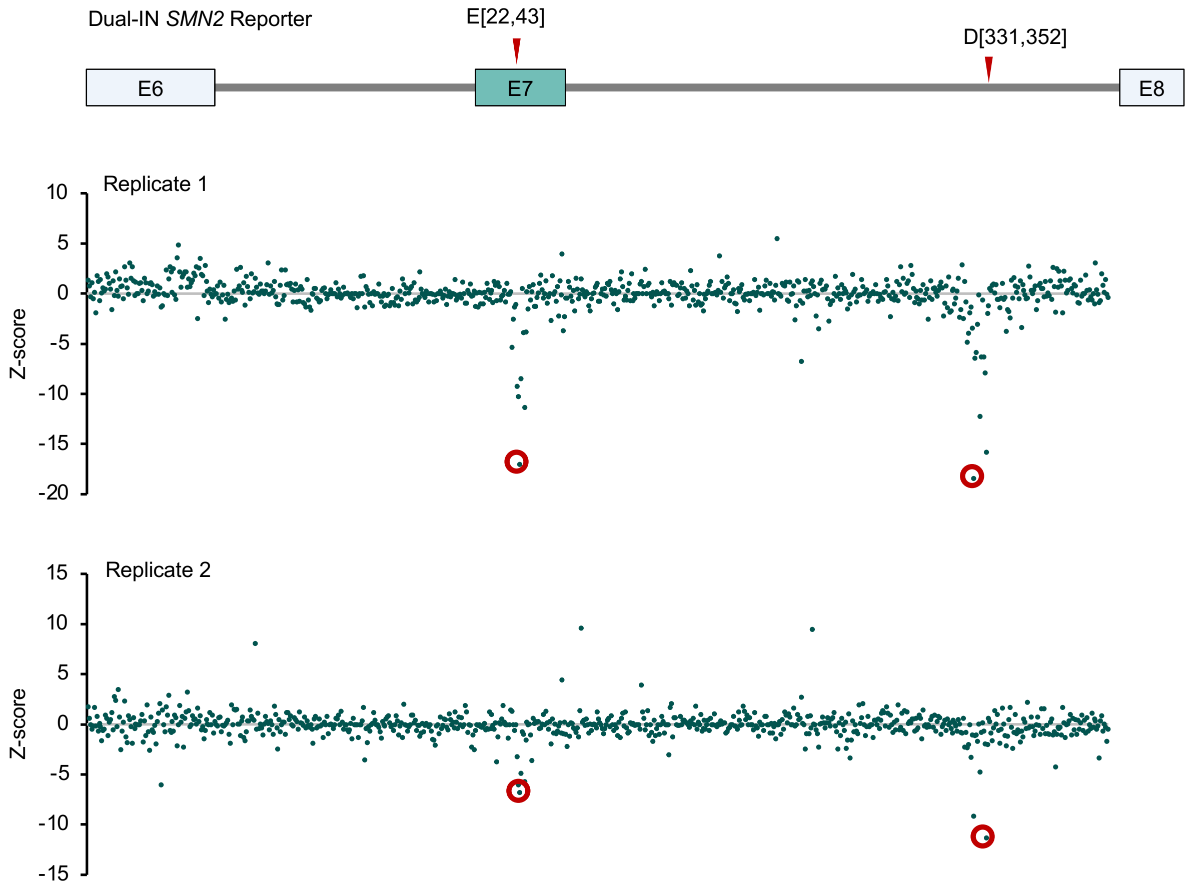

Supplementary Fig. 14: Screening results for the Dual-IN *SMN2* reporter for gRNAs of 22-nt. Related to Figure 3b in the main text. gRNA enrichment, as measured by a Z-score comparing cells in the top and bottom sorted bins based on tdTomato/eGFP fluorescence ratio to the unsorted cells, is plotted against the *SMN2* position (the first base pair corresponding to the 3' end of the gRNA spacer) for two independent replicates. Representative gRNAs targeting known SREs (E[22,43]) and a novel splicing enhancer in downstream intron (D[331,352]) are highlighted.

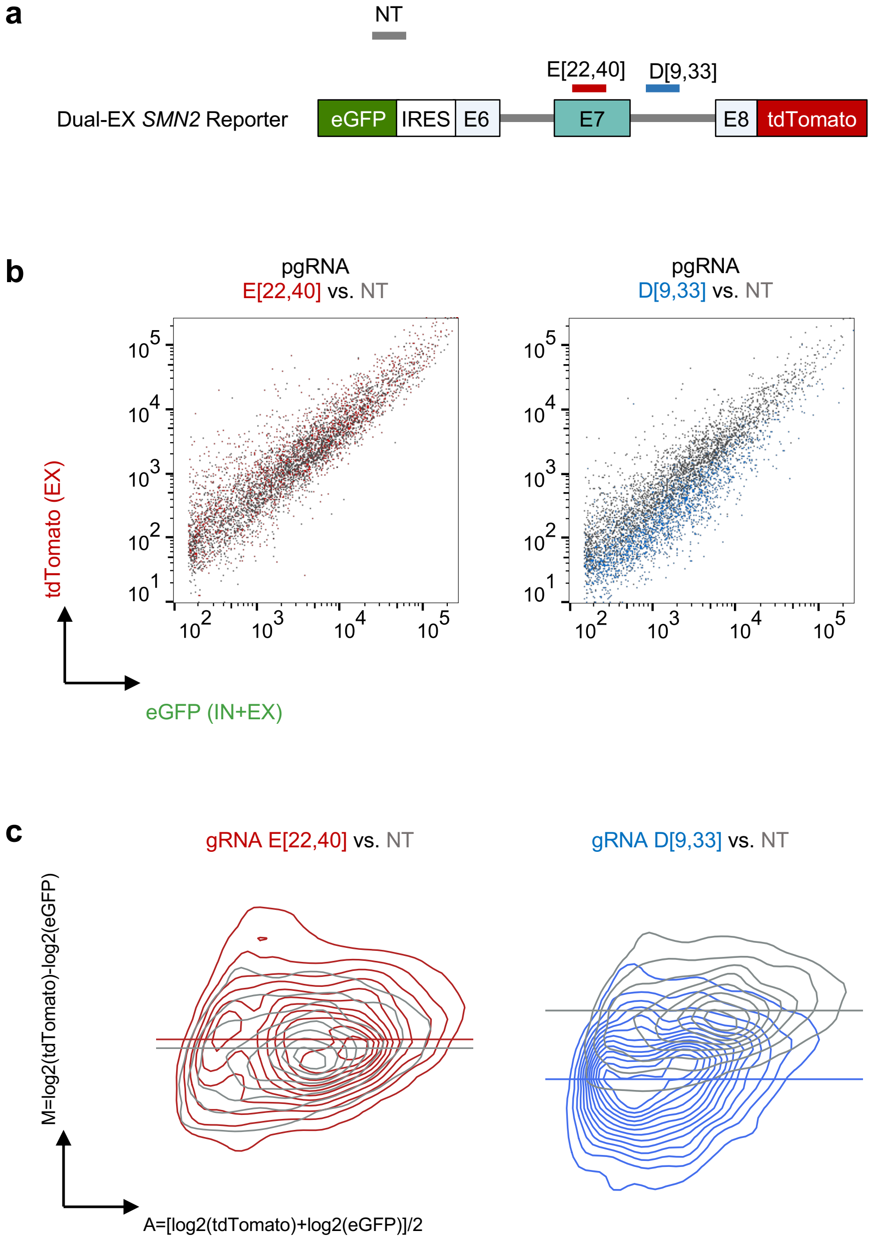

Supplementary Fig. 15: Splicing modulation using individual gRNAs targeting the Dual-EX *SMN2* splicing reporter via transfection. a, Schematic illustrating splicing-modulating gRNAs E[22,40] (red) and D[9,33] (blue) and NT control gRNA, which were individually transfected along with the plasmids expressing dCas13d-BFP and the Dual-EX *SMN2* reporter in HEK293T cells. **b,** FACS (b) and MA-contour plots (c) of tdTomato and eGFP fluorescence intensities in transfected cells. Datasets were downsampled to 2,475 BFP-positive events per sample.

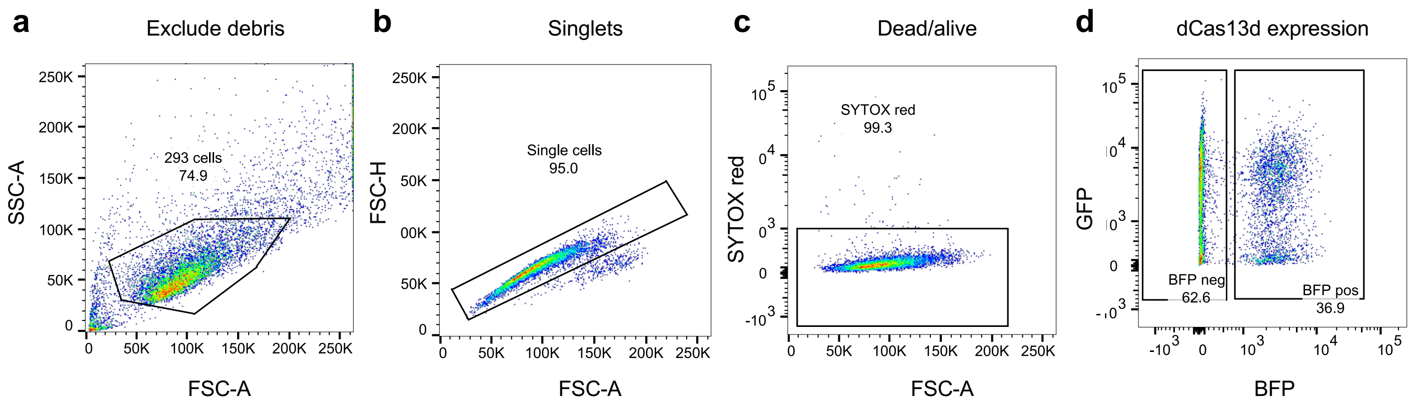

Supplementary Fig. 16: Gating scheme for Dual-EX *SMN2*/dCas13d-BFP cells transduced with a pooled lentivirus gRNA library. Cells with stable expression of DOX-inducible Dual-EX *SMN2* splicing reporter and dCas13d-BFP were transduced with the pooled lentivirus gRNA library. See Supplementary Fig. 2 legends for more details.

a

Dual-EX *SMN2* Samples	Total reads Replicate 1	Perfect gRNA Reads Replicate 1	Total reads Replicate 2	Perfect gRNA Reads Replicate 2
Unsorted	11,244,354	8,962,706 (79.7%)	13,958,424	10,797,580 (77.4%)
Top Bin BFP positive	13,777,536	11,049,081 (80.2%)	11,121,682	8,704,872 (78.3%)
Bottom Bin BFP positive	13,385,070	10,645,588 (79.5%)	15,691,199	12,139,831 (77.4%)
gRNAs not detected in unsorted	-	46 (2.4%)	-	54 (2.8%)

b

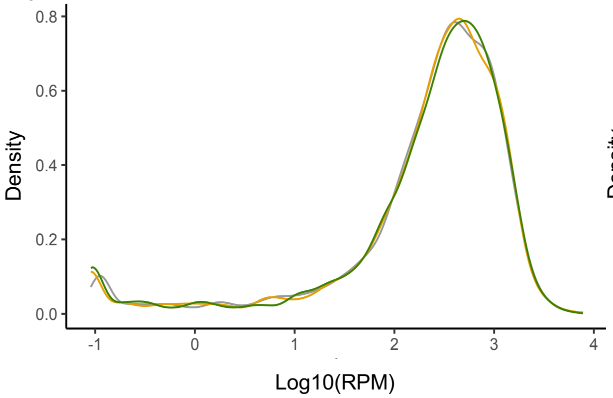

c

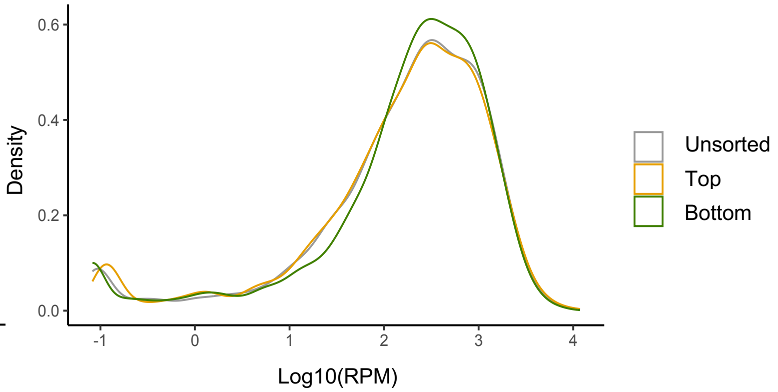

Supplementary Fig. 17: gRNA sequencing statistics for screening of Dual-EX *SMN2* splicing reporter. a, Number of total reads and perfectly matched gRNA reads in each sample from replicate 1 and replicate 2. **b,c**, Distribution of normalized read count, reads per million (RPM) of gRNAs for replicate 1 (b) and 2 (c).

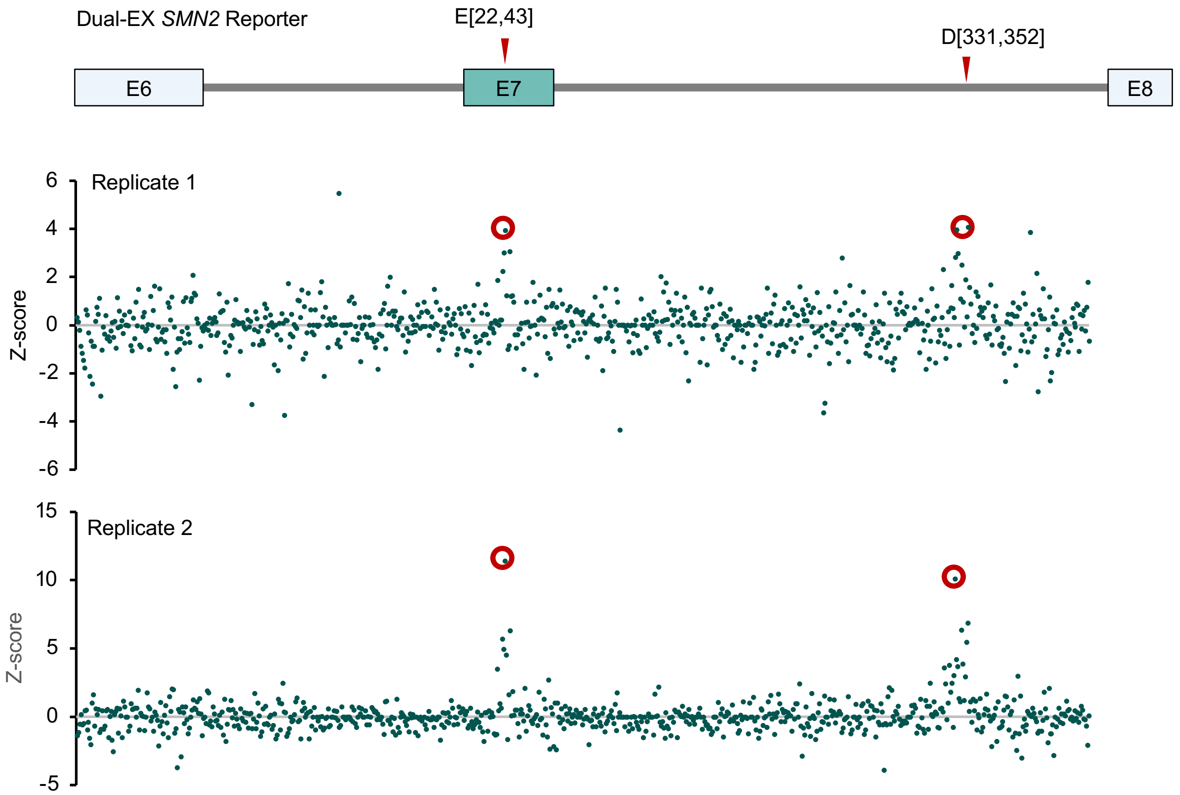

Supplementary Fig. 18: Screening results for the Dual-EX *SMN2* reporter for gRNAs of 22-nt. Related to Figure 4c in the main text.gRNA enrichment, as measured by a Z-score comparing cells in the top and bottom sorted bins based on tdTomato/eGFP fluorescence ratio to the unsorted cells, is plotted against the *SMN2* position (the first base pair corresponding to the 3' end of the gRNA spacer) for two independent replicates. Representative gRNAs targeting known SREs (E[22,43]) and a novel splicing enhancer in downstream intron (D[331,352]) are highlighted.

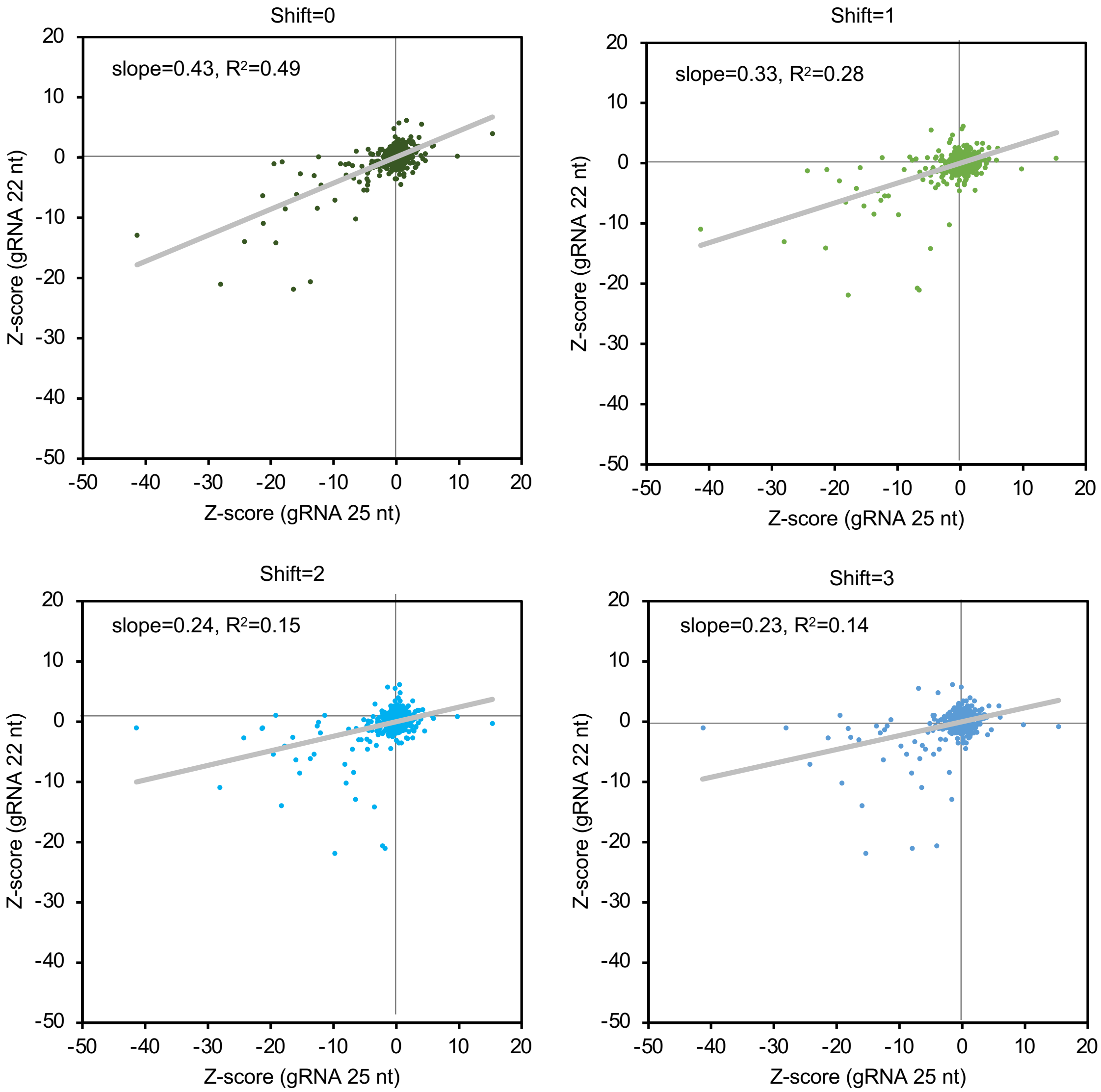

Supplementary Fig. 19: Comparison of gRNAs of different spacer lengths on splicing modulation. Related to Fig. 5 in the main text. The correlation of the Z-scores for 25-nt gRNA and 22-nt gRNA is plotted similarly as Fig. 5b in the main text, except that different shift comparison pairs were plotted separately. Each 25-nt gRNA is compared with four different 22-nt gRNAs that fully cover it with different start positions of the 22-nt gRNA. Shift 0 is defined when the compared 25-nt vs. 22-nt gRNAs have the same 5' end, while shifts 1, 2, and 3 indicate when the 22-nt gRNA is shifted for that number of corresponding nucleotides towards the 3' end (i.e., Shift 3 indicates the case when the two compared gRNAs coincide at the 3' end). The regression line and the slope and squared correlation (R^2) are also indicated.

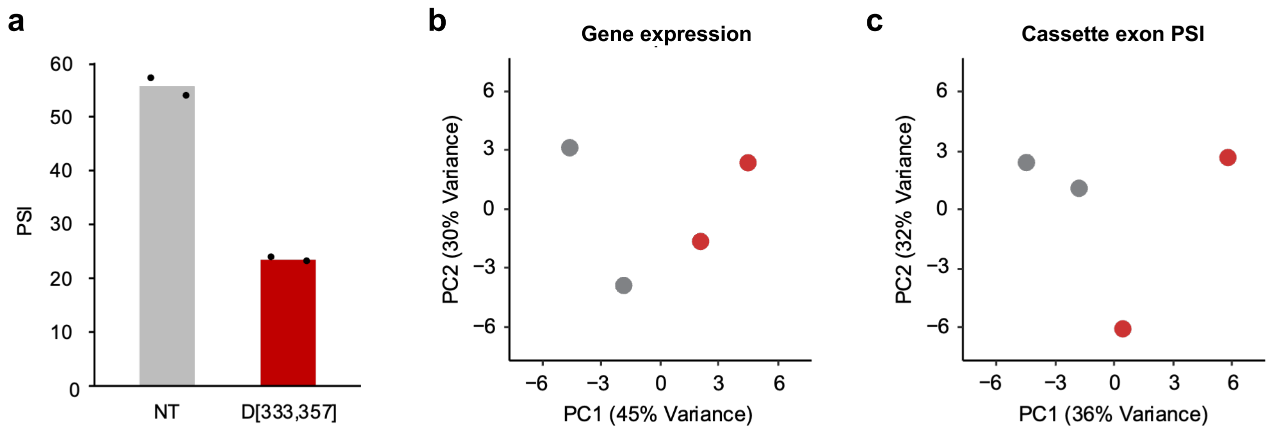

Supplementary Fig. 20: Transcriptomic analysis of gRNA-mediated splicing modulation. Bulk RNA-seq analysis was performed on HEK293T cells treated with NT gRNA (gray) or gRNA targeting the novel distal intronic silencer ISE-D1 D[333, 357] (red). **a,** Endogenous *SMN2* exon 7 inclusion levels in samples treated with NT and D[333,357] gRNAs. In HEK293T cells treated with D[333,357], *SMN2* exon 7 was significantly reduced ($p < 0.01$, n=2 per group, t-test). The mean exon inclusion level from two replicates of independent transfections is shown for each condition. **b,c,** Principal component analysis (PCA) on gene expression (b) and cassette splicing patterns (c) did not show consistent changes between groups at the whole transcriptomic level, suggesting minimal systematic off-target effects from gRNA-mediated splicing modulation.
