# Supplementary material for: CRISPR-dCas13d-based deep screening of proximal and distal splicing-regulatory elements
Source: Nat Commun. 2024 May 7;15:3839. doi: 10.1038/s41467-024-47140-8 (PMC11076525; doi:10.1038/s41467-024-47140-8)
Supplement: Supplementary file 3 — Description of Additional Supplementary Information [file 41467_2024_47140_MOESM3_ESM.pdf]

**Description of Additional Supplementary Information**

File Name: Supplementary Data 1

Description: List of oligo and primer sequences.

File Name: Supplementary Data 2

Description: List of gRNAs in the SMN2 library.

File Name: Supplementary Data 3

Description: Summary of cell sorting numbers in all experiments described in the study.

File Name: Supplementary Data 4

Description: Summary of screen results of the SMN2 library. P-values were calculated from the Z-score based on normal distribution (two-sided), followed by the calculation of false discovery rate (FDR) using the Benjamini–Hochberg procedure.
