# Supplementary material for: CRISPR-dCas13d-based deep screening of proximal and distal splicing-regulatory elements
Source: Nat Commun. 2024 May 7;15:3839. doi: 10.1038/s41467-024-47140-8 (PMC11076525; doi:10.1038/s41467-024-47140-8)
Supplement: Supplementary file 5 — Reporting Summary [file 41467_2024_47140_MOESM5_ESM.pdf]

nature portfolio

Corresponding author(s): Chaolin Zhang

Last updated by author(s): 02/29/2024

Reporting Summary

Nature Portfolio wishes to improve the reproducibility of the work that we publish. This form provides structure for consistency and transparency in reporting. For further information on Nature Portfolio policies, see our Editorial Policies and the Editorial Policy Checklist.

Statistics

For all statistical analyses, confirm that the following items are present in the figure legend, table legend, main text, or Methods section.

n/a	Confirmed	
☐	☒	The exact sample size (*n*) for each experimental group/condition, given as a discrete number and unit of measurement
☐	☒	A statement on whether measurements were taken from distinct samples or whether the same sample was measured repeatedly
☐	☒	The statistical test(s) used AND whether they are one- or two-sided<br>*Only common tests should be described solely by name; describe more complex techniques in the Methods section.*
☐	☒	A description of all covariates tested
☐	☒	A description of any assumptions or corrections, such as tests of normality and adjustment for multiple comparisons
☐	☒	A full description of the statistical parameters including central tendency (e.g. means) or other basic estimates (e.g. regression coefficient) AND variation (e.g. standard deviation) or associated estimates of uncertainty (e.g. confidence intervals)
☐	☒	For null hypothesis testing, the test statistic (e.g. *F*, *t*, *r*) with confidence intervals, effect sizes, degrees of freedom and *P* value noted<br>*Give P values as exact values whenever suitable.*
☒	☐	For Bayesian analysis, information on the choice of priors and Markov chain Monte Carlo settings
☒	☐	For hierarchical and complex designs, identification of the appropriate level for tests and full reporting of outcomes
☐	☒	Estimates of effect sizes (e.g. Cohen's *d*, Pearson's *r*), indicating how they were calculated

Our web collection on statistics for biologists contains articles on many of the points above.

Software and code

Policy information about availability of computer code

Data collection	No custom code was used for data collection.
Data analysis	FlowJo was used for FACS data analysis. For Illumina sequencing data from the screening, cutadapt 3.4 was used for adapter trimming, the python script count_spacers.py (ref. 74) was used for gRNA counting. Downstream statistical analysis was performed using custom scripts available at https://github.com/chaolinzhanglab/rush. RNA-seq data were processed analyzed using OLego (v1.1.5) and the Quantas analysis pipeline (v1.1.1). See Method section for a detailed description.

For manuscripts utilizing custom algorithms or software that are central to the research but not yet described in published literature, software must be made available to editors and reviewers. We strongly encourage code deposition in a community repository (e.g. GitHub). See the Nature Portfolio guidelines for submitting code & software for further information.

Data

Policy information about availability of data

All manuscripts must include a data availability statement. This statement should provide the following information, where applicable:

- Accession codes, unique identifiers, or web links for publicly available datasets
- A description of any restrictions on data availability
- For clinical datasets or third party data, please ensure that the statement adheres to our policy

Illumina sequencing data from the SMN2 splicing screens and RNA-sequencing have been deposited to NCBI Short Read Archive (SRA; accession number:

PRJNA1052769). Plasmids will be deposited to Addgene. Other data and materials that support the findings of this research are available from the corresponding author upon request.

Research involving human participants, their data, or biological material

Policy information about studies with human participants or human data. See also policy information about sex, gender (identity/presentation), and sexual orientation and race, ethnicity and racism.

Reporting on sex and gender	n/a
Reporting on race, ethnicity, or other socially relevant groupings	n/a
Population characteristics	n/a
Recruitment	n/a
Ethics oversight	n/a

Note that full information on the approval of the study protocol must also be provided in the manuscript.

Field-specific reporting

Please select the one below that is the best fit for your research. If you are not sure, read the appropriate sections before making your selection.

☒ Life sciences ☐ Behavioural & social sciences ☐ Ecological, evolutionary & environmental sciences

For a reference copy of the document with all sections, see nature.com/documents/nr-reporting-summary-flat.pdf

Life sciences study design

All studies must disclose on these points even when the disclosure is negative.

Sample size	All screens (Dual-IN and Dual-EX) have been conducted in two replicate experiments. A gRNA library of 1,937 was tested. All transfection experiments have been conducted in two or more independent biological replicate transfections. The sample size was determined by following common practices for similar high-throughput screenings.
Data exclusions	For the screen, Z-scores were filtered to keep only those gRNAs with a read count≥5 in both compared conditions and RPM≥20 in at least one of the two compared conditions.
Replication	All screens (Dual-IN and Dual-EX) have been conducted in two replicate experiments. All transfection experiments have been conducted in two or more independent biological replicate transfections. The screen findings were confirmed and validated in more than two replicates.All replicates were included in the data presented in this study.
Randomization	n/a. We followed the common practices for similar studies.
Blinding	There was no blinding in the datasets we used. We followed the common practices for similar studies.

Reporting for specific materials, systems and methods

We require information from authors about some types of materials, experimental systems and methods used in many studies. Here, indicate whether each material, system or method listed is relevant to your study. If you are not sure if a list item applies to your research, read the appropriate section before selecting a response.

Materials & experimental systems

n/a	Involved in the study	
☐	☒	Antibodies
☐	☒	Eukaryotic cell lines
☒	☐	Palaeontology and archaeology
☒	☐	Animals and other organisms
☒	☐	Clinical data
☒	☐	Dual use research of concern
☒	☐	Plants

Methods

n/a	Involved in the study	
☒	☐	ChIP-seq
☐	☒	Flow cytometry
☒	☐	MRI-based neuroimaging

Antibodies

Antibodies used

primary antibody mouse anti-HA (Sigma-Aldrich, H9658, 1:2,000); mouse anti-alpha-tubulin (Sigma-Aldrich, T6074, 1:10,000); goat anti-mouse secondary (Thermo Fisher 31436; 1:10,000 dilution)

Validation

Antibodies were validated by the vendors and previous studies.

Eukaryotic cell lines

Policy information about cell lines and Sex and Gender in Research

Cell line source(s)

HEK293T (CRL-3216) cells were obtained from American Type Culture Collection (ATCC). Flp-In T-REx 293; R78007 was obtained from ThermoFisher.

Authentication

All cell lines used have been authenticated by the original vendors using techniques such as STR profiling.

Mycoplasma contamination

Cell lines were not tested for mycoplasma contamination.

Commonly misidentified lines
(See ICLAC register)

No commonly misidentified cell lines were used.

Flow Cytometry

Plots

Confirm that:

☒ The axis labels state the marker and fluorochrome used (e.g. CD4-FITC).

☒ The axis scales are clearly visible. Include numbers along axes only for bottom left plot of group (a 'group' is an analysis of identical markers).

☒ All plots are contour plots with outliers or pseudocolor plots.

☒ A numerical value for number of cells or percentage (with statistics) is provided.

Methodology

Sample preparation

Cells were harvested 48 h post-transfection for flow cytometry to analyze the splicing levels with fluorescence. Cells were washed with PBS, detached with trypsin 0.05% (Corning 25-052-Cl), and resuspended in cold PBS after centrifugation at 500 RCF for 3 minutes. Resuspended cells were filtered through a 35 µM cell strainer and stained with either propidium iodide (Sigma-Aldrich P4864) or SYTOX deep red stain (Thermo Scientific, S11380) for dead/alive cells.

Instrument

Flow cytometry data acquisition and sorting were performed on a BD FACS Aria III.

Software

BD FACSDiva software was used to collect the data; FlowJo was used to analyze the data.

Cell population abundance

A minimum of 2E106 cells were collected for the unsorted sample, estimated at 1,000x coverage for the initial input gRNAs. For the sort, BFP-positive cells were fractionated based on tdTomato/eGFP ratio with the top 5% and bottom 5% populations collected. To ensure ~2,800x gRNA coverage of the collected bins, a minimum of 280,000 cells were collected for each bin (Supplementary Table 3).

Gating strategy

For FACS analysis, cells were gated by forward and side scatter to remove potential multiplets. Cells were additionally gated with a live-dead staining. For relevant experiments, BFP expression was further selected. For each sample we analyzed at least 2,400 cells. For transfection experiments, the gating strategy is showing in Supplementary Figure 2 a-d. For the gRNA library screens, the gating strategies are shown in Supplementary Figure 12 and Supplementary Figure 16.

☒ Tick this box to confirm that a figure exemplifying the gating strategy is provided in the Supplementary Information.
